# *Euterpe edulis* Extract but Not Oil Enhances Antioxidant Defenses and Protects against Nonalcoholic Fatty Liver Disease Induced by a High-Fat Diet in Rats

**DOI:** 10.1155/2016/8173876

**Published:** 2016-06-23

**Authors:** Rodrigo Barros Freitas, Rômulo Dias Novaes, Reggiani Vilela Gonçalves, Bianca Gazolla Mendonça, Eliziária Cardoso Santos, Andréia Queiroz Ribeiro, Luciana Moreira Lima, Luciano Gomes Fietto, Maria do Carmo Gouveia Peluzio, João Paulo Viana Leite

**Affiliations:** ^1^Department of Biochemistry and Molecular Biology, Federal University of Viçosa, 36570-000 Viçosa, MG, Brazil; ^2^Department of Structural Biology, Federal University of Alfenas, 37130-000 Alfenas, MG, Brazil; ^3^Department of Animal Biology, Federal University of Viçosa, 36570-000 Viçosa, MG, Brazil; ^4^School of Medicine, Federal University of Jequitinhonha and Mucuri Valleys, 39100-000 Diamantina, MG, Brazil; ^5^Department of Nutrition and Health, Federal University of Viçosa, 36570-000 Viçosa, MG, Brazil; ^6^Department of Medicine and Nursing, Federal University of Viçosa, 36570-000 Viçosa, MG, Brazil

## Abstract

We investigated the effects of* E. edulis* bioproducts (lyophilized pulp [LEE], defatted lyophilized pulp [LDEE], and oil [EO]) on nonalcoholic fatty liver disease (NAFLD) induced by a high-fat diet (HFD) in rats. All products were chemically analyzed.* In vivo*, 42 rats were equally randomized into seven groups receiving standard diet, HFD alone or combined with EO, LEE, or LDEE. After NAFLD induction, LEE, LDEE, or EO was added to the animals' diet for 4 weeks. LEE was rich in polyunsaturated fatty acids. From LEE degreasing, LDEE presented higher levels of anthocyanins and antioxidant capacity* in vitro*. Dietary intake of LEE and especially LDEE, but not EO, attenuated diet-induced NAFLD, reducing inflammatory infiltrate, steatosis, and lipid peroxidation in liver tissue. Although both* E. edulis* bioproducts were not hepatotoxic, only LDEE presented sufficient benefits to treat NAFLD in rats, possibly by its low lipid content and high amount of phenols and anthocyanins.

## 1. Introduction

Nonalcoholic fatty liver disease (NAFLD) has increased worldwide and has been widely associated with metabolic diseases such as obesity and dyslipidemia [1, 2]. Although the pathogenesis of NAFLD is complex and multifactorial, lifestyle, especially unhealthy dietary habits, is a pivotal risk factor for this condition [3–5]. NAFLD has become the most common liver disease in the United States and developed countries [1, 3, 5]. It is recognized that NAFLD develops as a consequence of immune-inflammatory mechanisms, which trigger the overproduction of reactive oxygen species (ROS) and reactive liver damage [1, 6]. In the absence of effective treatments, some patients with NAFLD may progress to nonalcoholic steatohepatitis (NASH), cirrhosis, hepatocellular carcinoma, liver failure, and death [2, 4].

In the last decades, foods rich in flavonoids and anthocyanins have been marketed as nutraceuticals or functional foods [7–9], whose therapeutic potential is often claimed, but mostly unproven [10]. A broad spectrum of biological activity has been attributed to polyphenols. However, particularly their immunomodulatory (i.e., anti-inflammatory) and antioxidant properties, as well as the alleged “absence of side effects,” have contributed to the wide acceptance of such substances in daily health care [10, 11]. Since* in vitro* and* in vivo* evidences of the therapeutic efficiency and biosafety of dietary intake of polyphenol-based products are frequently conflicting [10, 12, 13], to determine whether polyphenol supplementation could be useful for treating metabolic disturbances, including NAFLD, represents a challenging task.

Considering that polyphenols can modulate inflammation, oxidative stress, and energy metabolism [12–14], the rational screening of vegetable extracts rich in polyphenols has been considered a promising strategy for the development of new products potentially applicable in the management of metabolic disorders [7, 12, 14]. The fruit of the palm* Euterpe edulis* Mart. (Arecaceae), also called “açaí,” has emerged as an important source of natural antioxidants, especially anthocyanins [13, 15–17]. Recently, our research group identified a high content of anthocyanins in an* E. edulis* extract and a remarkable antioxidant potential* in vitro* [13, 17]. Furthermore, dietary intake of* E. edulis* extract alone [17] or combined with aerobic exercise training [13] was effective in attenuating genetically determined dyslipidemia and steatosis in apolipoprotein E knockout mice (ApoE^−/−^) fed a normocaloric diet [17]. However, the biological potential of* E. edulis* pulp and its derivatives to treat environmental dyslipidemia and NAFLD (determined by dietary intake)* in vivo* and its applicability as a functional food still needs to be proven. Thus, in the present study, we produced three derivatives of* E. edulis* fruit (lyophilized pulp, defatted lyophilized pulp, and pulp oil) and investigated the effect of dietary supplementation with these derivatives on morphofunctional liver injury induced by a high-fat diet in rats. We specifically hypothesized that supplementation with* E. edulis* derivatives, especially the extract rich in phenols and anthocyanins, would protect liver tissue against NAFLD.

## 2. Methods and Materials

### 2.1. Plant Material and Extraction of LEE, LDEE, and EO

*E. edulis* fruit was collected in an Atlantic Forest biome (latitude: −20, 97337439; longitude: −42, 52883943; height: 744.661 m), Brazil. Ripe fruits were selected, weighed, and disinfected with chlorinated water. The pulp was separated in an industrial depulper, passed through a fine mesh screen, and lyophilized (Liotop lyophilizer, Brazil). The lyophilized pulp of* E. edulis* was named LEE. An LEE sample was subjected to oil extraction by a defatting process (18.0 g of LEE was extracted with 600 mL ethyl ether using a Soxhlet extractor for 12 h). The lipophilic solvent was completely removed by rotary evaporator, producing* E. edulis* fruit oil (EO). The remaining defatted lyophilized extract of* E. edulis* fruit was named LDEE.

### 2.2. LEE and LDEE Centesimal Composition

The extracts were analyzed for moisture, ash, lipid, protein, and carbohydrate according to the official methods described by the Association of Official Analytical Chemists (AOAC) [18]. Total lipids were quantified after extraction with ether in the Soxhlet extractor. The total protein content was measured by the classic Kjeldahl method, and carbohydrate content was determined by percentage difference by subtracting the values of moisture, ash, lipids, and proteins [18]. The levels of alimentary soluble and insoluble fiber were determined by a gravimetric-enzymatic method [19] using a commercial kit (Sigma-Aldrich, Brazil). Fibers fractionation was performed according to the Van Soest method [19]. The estimated percentage of cell wall (cellulose, hemicellulose, and lignin) is given by the sum of the neutral detergent-soluble fractions. The less digestible portion of the cell wall (cellulose and lignin) was estimated by analysis of fiber in acid detergent in the insoluble fraction.

### 2.3. Spectrophotometric Determination of Phenolic Compounds and Anthocyanins in LEE and LDEE

The total phenolic content was determined with the Folin-Ciocalteu method using gallic acid as the standard [20]. The absorbance at 765 nm was evaluated by spectrophotometry. The results were expressed in mg of gallic acid equivalent per gram of dry extract (mg GAE/g). The total anthocyanin content (TAC) was determined according to Cheng and Breen [21]. The absorbance of the mixtures was measured at 520 and 700 nm in buffers at pH 1.0 and 4.5. TAC (100 g/g of sample) was calculated using the equation for cyanidin-3-glucoside: TAC (%  w/w) = *A* × MW × DF × *V* × 100%/*ε* × *lW*, where *A* = (*A*_520_ − *A*_700_)_pH 1.0_ − (*A*_520_ − *A*_700_)_pH 4.5_, MW (cyanidin-3-glucoside molecular weight) = 449.2 g/mol, DF is dilution factor (0.4), *ε* (cyanidin-3-glucoside extinction coefficient) = 26.900 mol/L, *l* is optical path in cm, *W* is weight of sample (mg), and 103 is conversion factor from g to mg.

### 2.4. Determination of LEE and LDEE Antioxidant Activity

The antioxidant activity of LEE and LDEE was determined* in vitro* by the stable organic free radical DPPH (2,2-diphenyl-1-picrylhydrazyl) photocolorimetric method [22]. DPPH reagent (Sigma-Aldrich, USA) was resuspended with methanol to obtain a DPPH working solution at 0.06 mM or 60 *μ*M. Solutions were prepared from LEE and LDEE in different concentrations (0.1 to 25.0 mg/mL). An aliquot of each solution (0.1 mL) was added to DPPH solution (0.06 mM). The solutions were read in a spectrometer at 515 nm and DPPH radical scavenging activities were calculated: % DPPH radical scavenging = ([*A*_control_ − *A*_sample_]/*A*_control_) × 100.

### 2.5. Analysis of Tocopherols and Tocotrienols in LEE, LDEE, and EO

The extraction and analysis of vitamin E components (*α*-, *β*-, *γ*-, and *δ*-tocopherols and tocotrienols) were performed according to Cardoso et al. [23]. The vitamin E components were analyzed by HPLC using 15 *μ*L LDEE, 10 *μ*L LEE, and 5 *μ*L EO. Peak identification was based on comparison of RT values with six authentic standards of tocopherols and tocotrienols. The quantification was performed by an analytical curve constructed from injection of six increasing concentrations of standard solutions. The total content of vitamin E was calculated by adding the components of the vitamin E identified in the samples.

### 2.6. Fatty Acids Profile in EO

The fatty acid composition of EO was determined by gas chromatography according to the protocols described by AOAC [18].

### 2.7. *In Vivo* Assay

Male Wistar rats (30 days old, weighing 85 ± 5 g) were used. The animals were maintained under controlled conditions with light/dark periods of 12/12 h, temperature set to 21 ± 2°C, and relative air humidity of 60–70%. All experiments were approved by the Ethics Committee for Animal Research of the Federal University of Viçosa (protocol 41/2014).

### 2.8. Experimental Design and Diets

Forty-two animals were randomly assigned to seven groups with six animals each as follows: (1) HFD alone or combined with (2) 4% EO; (3) and (4) 5% or 10% LEE; (5) and (6) 5% or 10% LDEE; (7) control animals receiving a standard diet. Animals in groups G1 to G6 received a high-fat diet (HFD, cafeteria diet) for 4 weeks to induce hepatic damage [6]. After this period, LEE, LDEE, or EO was added to the animals' diet for another 4 weeks. Animals in G7 (control group) were concurrently treated with a standard diet for rodents (Presence, Paulínia, SP, Brazil). The HFD consisted of ham pâté, mortadella, bacon, chocolate, powdered milk, potato chips, and a commercial diet for rats (2 : 1 : 1 : 1 : 1 : 1 : 1) totaling 50% fat, 20% protein, and 30% carbohydrate [6]. All the diets and water were provided* ad libitum*. During the experiment, animals were weighed every 3 days and food intake was measured daily. After the experimental period, the animals were euthanized by cardiac puncture under anesthesia in a halothane chamber.

### 2.9. Histopathology and Stereology

Liver samples (median lobe) were fixed in Bouin's solution and dehydrated ethanol and embedded in paraffin. Histological sections (4 *μ*m thick) were cut and stained with hematoxylin and eosin (H&E). Morphological changes in liver tissue were examined by the stereological method, using a test system of 300 points in a standard test area (At) of 73 × 10^3^ *μ*m^2^. Sixty histological fields from each group (×400 magnification) were randomly sampled and a total of 4.65 × 10^4^ *μ*m^2^ liver area was analyzed. To avoid repeated analysis of the same histological area, sections were evaluated in semiseries, using one of every 20 sections. The volume densities of hepatocytes (*V*_v_ [hep], %), interstitium (*V*_v_ [int], %), sinusoid capillaries (*V*_v_ [inf], %), inflammatory cells (*V*_v_ [inf cells], %), and lipid droplets (*V*_v_ [LD], %) were estimated [24]. The volume densities (*V*_v_) were estimated by counting points using the following formula: *V*_v_ = *P*_P_ [structure]/*P*_T_, where *P*_P_ is the number of points situated over the structure of interest and *P*_T_ is the total test points of the test system [25, 26]. The volumes of 50 hepatocyte nuclei (*V* [HN], *μ*m^3^) for each animal were determined according to the kariometric method previously described [27]. All morphological analyses were performed using Image Pro-Plus 4.5 image analysis software (Media Cybernetics, Silver Spring, MD, USA) [26, 28].

### 2.10. Oxidative Stress

Lipid peroxidation in liver tissue was evaluated by measuring thiobarbituric acid reactive substances (TBARS) [29]. Briefly, a liver sample (100 mg) was homogenized in phosphate buffer (pH 7.0) and then centrifuged (10000 ×g, 10 min) and the supernatant was reacted with thiobarbituric acid solution (trichloroacetic acid 15%, thiobarbituric acid 0.375%, and 0.25 N HCl) for 15 min. The formation of TBARS was spectrophotometrically monitored at 535 nm as previously described.

Catalase (CAT) activity was evaluated in liver supernatant according to the method described by Aebi [30] by measuring the rate of H_2_O_2_ decomposition over 1 min. Glutathione-s-transferase (GST) activity was determined spectrophotometrically by the product formed from the complexation of reduced glutathione with 1-chloro-2,4-dinitrobenzene according to Keen et al. [31]. SOD activity was estimated by a xanthine oxidase method based on the production of hydrogen peroxide (H_2_O_2_) and the reduction of nitroblue tetrazolium [32].

### 2.11. Biochemical Parameters

Blood samples were collected and centrifuged, and the serum was used for the biochemical determination of ultrasensitive C-reactive protein (CRP), total cholesterol, triacylglycerol, alanine aminotransferase (ALT), and aspartate aminotransferase (AST). All parameters were analyzed using commercial kits according to the manufacturer's directions (Human* In Vitro* Diagnostics, Minas Gerais, Brazil) [24, 26, 28].

### 2.12. Statistical Analysis

The data were expressed as means ± standard deviations (mean ± SD). The normality in data distribution was verified using the D'Agostino-Pearson test. The morphological data were submitted to the Kruskal-Wallis test and the biochemical data were analyzed by unifactorial one-way analysis of variance (one-way ANOVA) followed by the Student-Newman-Keuls (SNK)* post hoc* test for multiple comparisons. Statistical significance was set at *p* < 0.05.

## 3. Results

The levels of total fiber (soluble and insoluble) and carbohydrate were higher in LDEE (fiber, 59.58%; carbohydrate, 77.74%) compared to LEE (fiber, 44.81%; carbohydrate, 62.66%; *p* < 0.05). Protein content was low and similar in LEE (7.34%) and LDEE (8.88%; *p* > 0.05). The lipid content of LEE (25%) was adequately removed after the degreasing process. The resultant LDEE presented a dramatic reduction (−2134 kJ/100 g) in caloric content (LEE, 3214 kJ/100 g, versus LDEE, 1080 kJ/100 g, Table 1).

The total polyphenol and anthocyanin content was higher in LDEE compared to LEE (*p* < 0.05). After LEE degreasing, LDEE was enriched with 46.53% of anthocyanins (*p* < 0.05). The antioxidant activity of LDEE (1428 mg/L) was 52.72% higher compared to LEE (3020 mg/L, *p* < 0.05, Table 2).

The concentrations of *α*-, *β*-, and *γ*-tocopherol isomers identified in EO were higher than in LEE and LDEE (EO > LEE > LDEE). Total vitamin E levels and *α*-tocopherol activity were also higher in EO compared to LEE and LDEE (Table 3). The main fatty acids identified in EO were unsaturated (76.6%), with a preponderance of oleic acid (50.29%) and linoleic acid (24.24%, Table 4).

**Figure 1:**
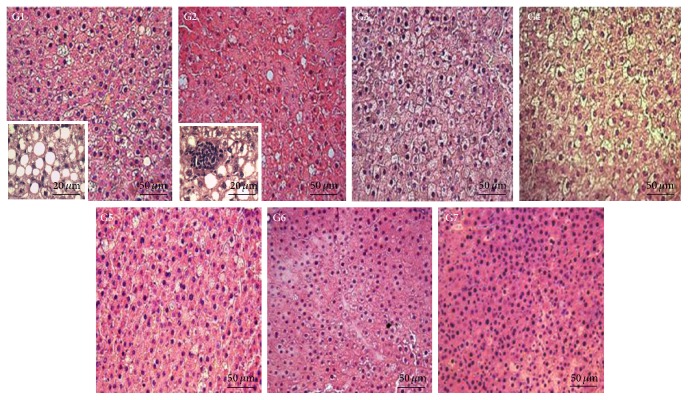
Representative photomicrographs of the liver tissue from rats treated with a high-fat diet (HFD) and the lyophilized extracts and oil of* E. edulis* fruits. LEE, lyophilized extract of* E. edulis* fruits; LDEE, defatted lyophilized extract of* E. edulis* fruits; EO,* E. edulis* oil. G1, HFD; G2, HFD + 4% EO; G3, HFD + 5% LEE; G4, HFD + 10% LEE; G5, HFD + 5% LDEE; G6, HFD + 10% LDEE; G7, commercial diet. The image highlighted in G1 indicates macrovesicular steatosis and in G2 inflammatory foci are additionally observed.

The HFD was effective in inducing marked liver steatosis in rats (G1). The control group (G7), which received a conventional diet, presented a normal liver structure with hepatocytes regularly organized in a cordonal pattern. No evidence of liver steatosis was observed in this group (Figure 1). All stereological parameters analyzed were completely impaired in G1 compared to G7 (*p* < 0.05, Table 5). These results indicated that animals in G1 presented intense parenchymal hypotrophy (*V*_v_ [hep] and* V* [HN]), tissue conjunctivalization (*V*_v_ [int]), and inflammatory infiltrate (*V*_v_ [inf]) and proved the accumulation of lipid droplets (*V*_v_ [LD]) in liver tissue (a pathognomonic feature of steatosis). In general, these parameters were similar in animals receiving EO and attenuated in those treated with 10% LDEE (G6) compared to G1 (*p* < 0.05). Supplementation with 10% LEE (G4) was also effective in reducing inflammatory infiltrate in liver tissue compared to G1 (*p* < 0.05).

**Figure 2:**
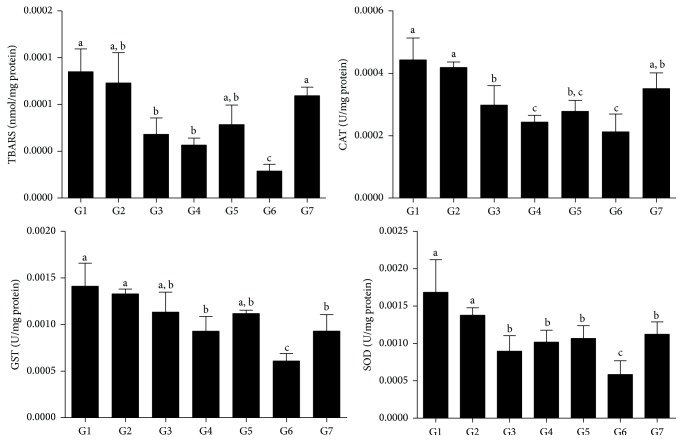
Markers of oxidative status in the liver tissue from rats treated with a high-fat diet (HFD) and the lyophilized extracts and oil of* E. edulis* fruits. MDA, malondialdehyde; CAT, catalase; GST, glutathione-s-transferase; SOD, superoxide dismutase. LEE, lyophilized extract of* E. edulis* fruits; LDEE, defatted lyophilized extract of* E. edulis* fruits; EO,* E. edulis* oil. G1, HFD; G2, HFD + 4% EO; G3, HFD + 5% LEE; G4, HFD + 10% LEE; G5, HFD + 5% LDEE; G6, HFD + 10% LDEE; G7, commercial diet. ^a, b, c^Different letters in columns denote statistic difference between the groups, *p* < 0.05.

In general, the animals that received* E. edulis* extracts (LEE [G3 and G4] and LDEE [G5 and G6]), but not EO (G2), exhibited reduced MDA levels in liver tissue compared to G1 (*p* < 0.05, Figure 2). CAT and GST activities were lower in groups G4 and G6, and SOD content was lower in all groups receiving LEE and LDEE, both compared to G1 (*p* < 0.05). The lowest results for MDA and antioxidant enzymes were observed in G6.

The results of the serum biochemical analysis are shown in Table 6. Total cholesterol serum levels in G6 and G7 were similar but reduced compared to G1, G2, and G3 (*p* < 0.05). The levels of CRP, AST, ALT, triacylglycerol, and HDL cholesterol were similar in all groups (*p* > 0.05).

## 4. Discussion

In this study, we investigated the chemical composition of three derivatives obtained from* E. edulis* fruit and their applicability to protect liver tissue against diet-induced NAFLD in rats. Our results showed that* E. edulis* pulp is rich in carbohydrates and lipids, which is the main determinant of the high energy value of this pulp. In addition, all* E. edulis* products (integral extract [LEE], degreased extract [LDEE], and vegetal oil [EO]) presented high levels of antioxidant metabolites. The lipid fraction of* E. edulis* fruit has a higher polyunsaturated fatty acid content and a lower saturated lipid content, which makes it suitable for human consumption [11]. Similar results for the fatty acid composition of* E. edulis* were found by Borges et al. [15] and Neida and Elba [33], who demonstrated the predominance of oleic and linoleic acids in* E. edulis* and* E. oleracea* fruits, respectively. Although the Amazon rainforest açaí (*E. oleracea* fruit) has excelled in the international market ($121.7 m a year in the USA) [34], the Atlantic Forest açaí (*E. edulis* fruit) is still a food resource little explored. However, from a nutritional point of view, the chemical composition of* E. edulis* [13, 15, 17] and* E. oleracea* is similar [33, 35–37], corroborating the use of* E. edulis* fruit as a valuable food resource.

Both derivatives EO and LEE were rich in vitamin E. Due to the content of polyphenols and anthocyanins, LEE and LDEE presented high antioxidant activity* in vitro*. The degreasing process of LEE proved to be effective in increasing polyphenols and anthocyanins, resulting in an enhanced antioxidant capacity of LDEE. This finding indicated that, as expected, the antioxidant capacity of* E. edulis* fruit is related to its polyphenolic compounds. In fact, in a recent study we also proved the high content of anthocyanins (301.4 mg/100 g dry extract, especially cyanidin-3-glucoside and cyanidin-3-rutinoside) in an* E. edulis* extract similar to LEE [17]. There is evidence that anthocyanins such as cyanidin-3-O-glucoside and cyanidin-3-O-rutinoside have high antioxidant activity against peroxyl, hydroxyl, and peroxynitrite radicals, an effect potentially associated with the antioxidant capacity of* E. oleracea* pulp* in vivo* [37].

When tested* in rats*, dietary intake of LEE and LDEE, but not EO, was effective in mitigating the development or severity of NAFLD in rats fed an HFD. The extracts, especially LDEE at the highest dose (10%), had a positive impact in reducing hepatocyte hypotrophy, inflammatory infiltrate, microvesicular steatosis, and lipid peroxidation in liver tissue (i.e., MDA). Lipid accumulation, inflammation, and oxidative stress are three pivotal interdependent events in NAFLD pathogenesis [1, 38]. There is evidence that this triad feeds itself in a cycle that worsens morphological and functional liver damage [1, 5, 38, 39]. As a result of inflammatory processes, the activation of leucocytes increases the production of oxidizing agents such as nitric oxide (NO), superoxide (O_2_^−^), and hydroxyl (OH^−^) radicals, which are known for amplifying oxidative damage and hepatic dysfunction, including steatosis [24, 25, 28]. Thus, antioxidants and anti-inflammatory drugs have been indicated as rational strategies to prevent and treat NAFLD [2, 5]. Previous studies showed that dietary intake of plant extracts rich in phenols and anthocyanins is capable of downregulating the oxidation of lipids and cell proteins, reducing the inflammatory process and the severity of liver steatosis [13, 14, 17]. The antioxidant activity of many fruits comes from the combined synergic actions of compounds such as phenols, flavonoids, carotenoids, and vitamins C and E [33, 40]. However, in fruits like açaí, polyphenols and anthocyanins are the main contributors to the antioxidant capacity [14, 17, 35].

As expected, the diet-induced NAFLD triggered liver oxidative stress, requiring a reactive upregulation of antioxidant enzymes. Considering a mechanistic approach, we investigated whether these extracts acted by a direct or indirect way to mitigate lipid peroxidation. In general, while the animals receiving HFD alone or combined with EO presented high MDA levels and increased activities of CAT, SOD, and GST, the animals treated with LEE and LDEE showed the opposite. Thus, these findings indicated that both extracts act in a direct way (independent of the endogenous antioxidant enzymatic system) to neutralize reactive metabolites and modulate the oxidative status. It is possible that, by effectively neutralizing free radicals, LEE and LDEE reduce the consumption of antioxidant enzymes in antioxidant defense processes. This effect is remarkable since the exhaustion of antioxidant enzymes by mass production of free radicals is a pivotal mechanism of tissue damage [24–26]. However, several plant extracts rich in phenol compounds also act by upregulating the expression and activity of antioxidant enzymes, modulating endogenous antioxidant defenses [24, 25, 36]. Although there is evidence that the major anthocyanin of* E. edulis* and* E. oleracea* pulp (cyanidin-3-glucoside) is effective in suppressing the activity of antioxidant enzymes in rats [37], this is a poorly understood issue that requires further investigation.

While LEE and LDEE showed beneficial results, EO dietary intake presented no effect on NAFLD. Thus, animals treated with EO and those receiving HFD alone had high and similar liver damage. Considering that the high activity of CAT, SOD, and GST was similarly observed in these animals, it is possible that the content of vitamin E in EO is not enough to restrict the oxidative stress associated with diet-induced NAFLD. Although previous studies have shown a high antioxidant potential of açaí oil [15, 16, 35], this effect seems not to be reproducible* in vivo*. Considering the complexity of the disease model applied here, the absence of antioxidant activity cannot be exclusively attributed to the chemical composition of EO. As HFD is a robust model of obesity and NAFLD in rats [6, 41], it is reasonable to assume that additional lipid intake provided by EO supplementation could not be beneficial to treating NAFLD. However, as with LEE and LDEE, EO intake presented no evidence of liver toxicity, since CRP, ALT, and AST serum levels [42] were similar in all groups. Besides no apparent toxicity, larger doses and periods of administration should be investigated before determining the safety level and applicability of these* E. edulis* derivatives as food supplements.

Taken together, our results indicated that* E. edulis* pulp is a useful source of natural extracts and oil with high nutritional or biological properties. As determined by chemical analysis, unsaturated lipids are the main components responsible for the high energy value of LEE. By removing LEE lipid content, its degreased derivative (LDEE) presented increased antioxidant capacity* in vitro*, which was determined by higher levels of phenols and anthocyanins. When tested* in vivo*, the antioxidant potentials of LEE and LDEE were independent of endogenous antioxidant enzymes. Moreover, dietary intake of LEE and especially LDEE, but not EO, proved to be beneficial in attenuating diet-induced NAFLD in rats, reducing hepatocyte hypotrophy, inflammatory infiltrate, steatosis, and lipid peroxidation in liver tissue. As no morphological or functional parameter associated with hepatic steatosis was aggravated, the dietary intake of* E. edulis* derivatives seems to be safe. However, further clinical studies are still required in order to determine whether* E. edulis* pulp and its derivatives can be used as dietary supplements in the management of NAFLD induced by HFD.

## Supplementary Material

The detailed composition and energy value of the experimental diets are described in the Supplementary Material.

## Figures and Tables

**Table 1:** Centesimal chemical composition of the lyophilized extracts of *E. edulis* fruits before and after degreasing.

Components	LEE (%)	LDEE (%)
Moisture	2.37 ± 0.40^a^	9.29 ± 0.80^b^
Ash	3.04 ± 0.10^a^	4.09 ± 0.20^b^
Protein	7.34 ± 0.60^a^	8.88 ± 1.10^a^
Total lipid	24.59 ± 1.50	—
Carbohydrates	62.66 ± 2.40^a^	77.74 ± 1.80^b^
Soluble fibers	1.80 ± 0.00^a^	2.39 ± 0.010^b^
Insoluble fibers	43.11 ± 0.20^a^	57.19 ± 0.30^b^
Lignin	9.2 ± 0.30^a^	9.49 ± 0.40^a^
Cellulose	6.53 ± 1.10^a^	10.31 ± 0.70^b^
Hemicellulose	15.51 ± 2.80^a^	24.25 ± 2.50^b^
Cutin	7.10 ± 0.80^a^	10.70 ± 0.50^b^

Caloric value (kJ/100 g)	3214^a^	1080^b^

LEE, lyophilized extract of *E. edulis* fruits; LDEE, defatted lyophilized extract of *E. edulis* fruits; —, absent component. Data are expressed as mean of five replicates ± standard deviation. ^a,b^Means followed by the same letter in the line do not differ statistically among themselves.

**Table 2:** Polyphenols, anthocyanins, and *in vitro* antioxidant capacity of lyophilized extracts and oil of *E. edulis *fruits.

Components	LEE	LDEE
Polyphenols (mg GAE/g)	4.10 ± 0.13^a^	4.95 ± 0.07^b^
Anthocyanins (mg GAE/g)	2130 ± 114^a^	3121 ± 139^b^
Antioxidant capacity (mg/L)	3020 ± 80.00^a^	1428 ± 66.00^b^

LEE, lyophilized extract of *E. edulis* fruits; LDEE, defatted lyophilized extract of *E. edulis* fruits. GAE, gallic acid equivalent. The results were expressed as mean of five replicates ± standard deviation. ^a,b^Means followed by the same letter in the line do not differ statistically among themselves.

**Table 3:** Vitamin E content in lyophilized extracts and oil of *E. edulis *fruits.

Components	LEE	LDEE	EO
*α*-tocopherol	32.17 ± 0.61^a^	2.10 ± 0.30^b^	140.45 ± 3.56^c^
*β*-tocopherol	1.50 ± 0.01^a^	0.11 ± 0.01^b^	7.10 ± 0.07^c^
*γ*-tocopherol	1.71 ± 0.01^a^	0.10 ± 0.01^b^	7.37 ± 0.03^c^
Total vitamin E^1^	35.38 ± 0.61^a^	2.31 ± 0.03^b^	154.92 ± 3.67^c^
*α*-tocopherol activity	33.09 ± 0.62^a^	2.16 ± 0.03^b^	144.74 ± 2.60^c^

LEE, lyophilized extract of *E. edulis* fruits; LDEE, defatted lyophilized extract of *E. edulis* fruits; EO, *E. edulis *oil. ^1^Obtained by summing the concentrations of all isomers, with a weight of 1 for *α*-tocopherol and 0.3 for *β*- and *γ*-tocopherol. The results were expressed as mean of five replicates ± standard deviation. ^a,b,c^Means followed by the same letter in the line do not differ statistically among themselves.

**Table 4:** Fatty acids profile of *E. edulis* fruits oil.

Specification	Content (%)	Retention time (min)
Palmitic acid (16:0)	21.30 ± 0.20	24.191
Behenic acid (22:0)	0.04 ± 0.04	34.785
Margaric acid (17:0)	0.02 ± 0.03	26.240
Arachidic acid (20:0)	0.06 ± 0.07	31.342
Stearic acid (18:0)	1.99 ± 0.00	27.833

∑*Unsaturated* *fatty* *acids*	*23.4*	

Linoleic acid (18:2^Δ9,12^)	24.24 ± 0.25	30.572
Palmitoleic acid (16:1^Δ9^)	1.16 ± 0.07	25.411
Gadoleic acid (20:1^Δ11^)	0.49 ± 0.45	32.486
Oleic acid (18:1^Δ9^)	50.29 ± 0.52	29.048
Margaroleic acid (17:1^Δ9^)	0.04 ± 0.02	27.370

∑*Saturated* *fatty* *acids*	*76.6*	

The results were expressed as mean ± standard deviation of five replicates and expressed in fresh base. Chromatographic conditions are according to the Association of Official Analytical Chemists [18].

**Table 5:** Stereological parameters of the liver tissue from rats treated with a high-fat diet (HFD) and the lyophilized extracts and oil of *E. edulis *fruits.

Groups	*V*_v_ [hep], %	*V*_v_ [int], %	*V*_v_ [cap], %	*V*_v_ [Inf], %	*V*_v_ [LD], %	*V* [HN], *µ*m^3^
G1	78.11 ± 2.07^a,b^	21.89 ± 2.07^a,b^	9.26 ± 1.70^a^	6.63 ± 1.27^a^	23.39 ± 3.66^a,b^	428.11 ± 27.31^a^
G2	75.15 ± 2.31^a^	24.85 ± 2.31^a^	9.58 ± 2.02^a^	6.97 ± 1.83^a^	25.18 ± 3.82^a^	412.25 ± 30.12^a^
G3	78.02 ± 1.93^a,b^	21.98 ± 1.93^a,b^	10.05 ± 1.77^a^	6.81 ± 1.66^a^	22.05 ± 3.27^a,b^	436.41 ± 22.62^a^
G4	78.39 ± 2.11^a,b^	21.61 ± 2.11^a,b^	10.73 ± 2.05^a^	4.50 ± 1.35^b^	24.12 ± 3.39^a^	505.37 ± 24.39^b^
G5	78.92 ± 2.55^a,b^	21.08 ± 2.55^a,b^	9.81 ± 1.62^a^	5.02 ± 1.42^a,b^	20.26 ± 3.18^b^	459.13 ± 25.18^a^
G6	79.75 ± 2.47^b^	20.25 ± 2.47^b^	9.67 ± 1.75^a^	4.01 ± 1.19^b^	19.07 ± 2.95^b^	512.58 ± 21.72^b^
G7	83.37 ± 1.89^c^	17.63 ± 1.89^c^	10.40 ± 1.14^a^	3.11 ± 1.03^c^	7.38 ± 2.71^c^	573.81 ± 20.13^c^

LEE, lyophilized extract of *E. edulis* fruits; LDEE, defatted lyophilized extract of *E. edulis* fruits; EO, *E. edulis *oil. *V*_v_, volume density; hep, hepatocytes; int, interstitium; cap, capillaries; inflammatory cells; LD, lipid droplets; HN, hepatocytes nuclei. G1, HFD; G2, HFD + 4% EO; G3, HFD + 5% LEE; G4, HFD + 10% LEE; G5, HFD + 5% LDEE; G6, HFD + 10% LDEE; G7, commercial diet. ^a,b,c^Different letters in columns denote statistic difference among the groups, *p* < 0.05.

**Table 6:** Serum biochemical parameters (U/L) of rats treated with a hypercholesterolemic diet and *E. edulis*.

	US-PCR	Glucose	TC	TG	HDL	AST	ALT	ALP
G1	0.30 ± 0.12^a^	194.00 ± 54.70^a,b^	57.33 ± 5.75^a^	89.66 ± 38.83^a^	33.16 ± 5.49^a^	172.16 ± 98.65^a^	55.00 ± 18.91^a^	121.66 ± 30.6^a^
G2	0.37 ± 0.17^a^	159.66 ± 12.29^a,b^	50.83 ± 5.70^a^	98.33 ± 19.45^a^	36.83 ± 4.07^a^	183.16 ± 89.49^a^	73.83 ± 29.90^a^	179.00 ± 73.26^a^
G3	0.66 ± 0.47^a^	158.00 ± 13.57^a,b^	53.50 ± 6.05^a^	91.83 ± 11.30^a^	35.16 ± 1.72^a^	123.00 ± 32.92^a^	59.50 ± 21.49^a^	170.16 ± 46.81^a^
G4	0.42 ± 0.15^a^	190.16 ± 20.12^a^	62.50 ± 11.23^a,c^	124.83 ± 44.52^a^	36.33 ± 5.20^a^	101.00 ± 23.09^a^	44.50 ± 10.82^a^	166.83 ± 48.14^a^
G5	0.57 ± 0.22^a^	196.83 ± 27.12^a^	57.66 ± 3.44^a,b^	122.33 ± 42.54^a^	31.83 ± 3.25^a^	113.00 ± 19.40^a^	44.50 ± 8.52^a^	143.33 ± 30.28^a^
G6	0.21 ± 0.11^a^	200.66 ± 22.90^a^	66.66 ± 8.28^b,c^	114.83 ± 65.48^a^	34.66 ± 3.55^a^	94.00 ± 25.04^a^	52.00 ± 39.37^a^	130.33 ± 46.24^a^
G7	0.36 ± 0.18^a^	139.66 ± 18.47^b^	69.50 ± 8.82^b,c^	75.66 ± 11.41^a^	37.16 ± 8.97^a^	137.33 ± 38.95^a^	51.33 ± 14.47^a^	60.50 ± 25.86^b^

US-PCR, ultrasensitive C-reactive protein; TC, total cholesterol; TG, triglycerides; HDL, high density lipoproteins; AST, alanine aminotransferase; ALT, aspartate aminotransferase, ALP, alkaline phosphatase. G1, CD; G2, CD + 4% OE; G3, CD + 5% LEE; G4, CD + 10% LEE; G5, CD + 5% LEDE; G6, CD + 10% LEDE; G7, commercial diet. ^a,b,c^Different letters in columns denote statistic difference between the groups, *p* < 0.05.

## Abbreviations

LEE: Lyophilized pulp
LDEE: Defatted lyophilized
EO: Pulp oil
HFD: High-fat diet
NAFLD: Nonalcoholic fatty liver disease
ALT: Alanine aminotransferase
AST: Aspartate aminotransferase
AOAC: Association of Official Analytical Chemists
DPPH: 2,2-Diphenyl-1-picrylhydrazyl
TAC: Total anthocyanin content
*V*_v_: Volume density
TBARS: Thiobarbituric acid reactive substances
CAT: Catalase
GST: Glutathione-s-transferase
SOD: Superoxide dismutase
H_2_O_2_: Hydrogen peroxide.
