# Supplementary material for: Euterpe edulis Extract but Not Oil Enhances Antioxidant Defenses and Protects against Nonalcoholic Fatty Liver Disease Induced by a High-Fat Diet in Rats
Source: Oxid Med Cell Longev. 2016 Jun 23;2016:8173876. doi: 10.1155/2016/8173876 (PMC4935907; doi:10.1155/2016/8173876)
Supplement: Supplementary file 1 — The detailed composition and energy value of the experimental diets are described in the Supplementary Material. [file 8173876.f1.doc]

**Table S1.** Composition of the standard commercial diet.

| Composition* | **Mass (g)** | **Energy value (kJ/100g)*** |
| --- | --- | --- |
| Protein | 189 | 316.31 |
| Lipid | 112 | 421.75 |
| Carbohydrate | 518 | 866.93 |
| Insoluble fibers | 24 | # |
| Mix mineral | 57 | # |
| Mix vitamin | 100 | # |
| **Energy value (kJ)** | **1000** | **1605** |

*The composition and energy value was determined by the diet manufacturer (Presence, Paulinea, SP, Brazil).

**Table S2.** Composition of the high-fat diet (cafeteria diet).

| Composition | **Mass (g)** | **Energy value (kJ/100g)*** |
| --- | --- | --- |
| Ham pâté | 223 | 1087.84 |
| Potato chips | 111 | 2271.91 |
| Bacon | 111 | 2481.11 |
| Mortadella | 111 | 1125.5 |
| Cornstarch cracker | 111 | 1648.5 |
| Chocolate | 111 | 1677.78 |
| Powdered milk | 111 | 2079.45 |
| Commercial chow | 111 | 1506.24 |
| **Total content** | **1000** | **1734.79** |

*The energy value was determined by the manufacturer of each product included in the diet.

**Table S3.** Composition of the high-fat diet (HFD) combined with *E. edulis* derivatives.

| Composition | **HFD + 4% EO** | **HFD + 5% LEE** | **HFD + 10% LEE** | **HFD + 5% LDEE** | **HFD + 10% LDEE** |
| --- | --- | --- | --- | --- | --- |
| Ham pâté (g) | 214.08 | 211.85 | 200.70 | 211.85 | 200.70 |
| Potato chips (g) | 106.56 | 105.45 | 99.90 | 105.45 | 99.90 |
| Bacon (g) | 106.56 | 105.45 | 99.90 | 105.45 | 99.90 |
| Mortadella (g) | 106.56 | 105.45 | 99.90 | 105.45 | 99.90 |
| Cornstarch cracker (g) | 106.56 | 105.45 | 99.90 | 105.45 | 99.90 |
| Chocolate (g) | 106.56 | 105.45 | 99.90 | 105.45 | 99.90 |
| Powdered milk (g) | 106.56 | 105.45 | 99.90 | 105.45 | 99.90 |
| Commercial chow (g) | 106.56 | 105.45 | 99.90 | 105.45 | 99.90 |
| *E. edulis* derivative (g)* | 40.00 | 50.00 | 100.00 | 50.00 | 100.00 |
| **Energy value (kJ)** | **3144.86** | **3255.05** | **4775.31** | **2188.05** | **2641.31** |

EO, *E. edulis* oil; LEE, LDEE, lyophilized pulp of *E. edulis*; LDEE, defatted lyophilized pulp of *E. edulis*. *Energy values were based on centesimal composition of *E. edulis* derivatives.
